# Paroxysmal Tonic Upgaze in Pediatric Patients With Down Syndrome: A Case Report

**DOI:** 10.1155/crpe/7858435

**Published:** 2026-10-17

**Authors:** Sierra Sanchez, Elise O’Neil, Stacy McConkey

**Affiliations:** ^1^ Department of Pediatrics AdventHealth Orlando Orlando Florida USA adventhealth.com; ^2^ Department of Pediatrics Loma Linda University School of Medicine Loma Linda California USA llu.edu

## Abstract

Paroxysmal Tonic Upgaze (PTU) is a paroxysmal movement disorder characterized by episodes of sustained upward deviation of the eyes, which can be misidentified as a seizure. There has been no definite association made between PTU and Down syndrome although Down syndrome has been associated with various other ocular and neurologic conditions. This case report describes three patients with Down syndrome who presented with seizure‐like activity and were ultimately diagnosed with PTU. Consideration of PTU in a differential diagnosis, especially in patients with a history of Down syndrome, is essential for accurately distinguishing potentially benign conditions from epilepsy and can help clinicians avoid misdiagnosis and unnecessary treatments.

## 1. Introduction

Paroxysmal Tonic Upgaze (PTU) is often defined as episodes of sustained conjugate upward deviation of the eyes with compensatory neck flexion, downbeating saccades with attempted downgaze, and normal horizontal eye movements [1, 2]. This rare phenomenon can occur in children, with onset typically occurring in those under 1 year of age. Episodes can be triggered by febrile illness, fatigue, or anxiety and can be relieved by sleep [1, 2]. PTU is diagnosed with an, otherwise, normal neurological examination, normal electroencephalogram (EEG) and neuroimaging, and eventual improvement [1, 2]. PTU can be misidentified as a seizure if clinicians are not aware of the phenomenon.

Between its description in 1988 and 2002, 49 PTU cases were reported and linked to autosomal dominant and recessive disorders, structural lesions, fetal exposure to sodium valproate, and pinealoma [2]. An association with the chromosomal disorder Beckwith–Widmann syndrome and abnormalities on chromosome 15 have also been described. More recently, the benign nature of PTU has been challenged. In one recent study of paroxysmal disorders, eight patients were diagnosed with PTU (referred to in the article as BTU; benign tonic upward gaze) and 62% had some form of cognitive defects [3]. There was also a 44% rate of *CACNA1A* mutation, which has previously been associated with PTU [3]. Other studies have shown up to a 69% association with developmental delay [4]. Thus far, PTU has never been associated with Down syndrome in published literature [3].

Down syndrome has been associated with a variety of other ocular findings including infantile nystagmus, strabismus, amblyopia, cataracts, refractive and accommodation errors, and glaucoma [3, 5]. Patients with Down syndrome also can present with many associated neurological conditions. All the following conditions have a prevalence of 10% or greater in this population: hypotonia, intellectual disability, hearing impairment, atlantoaxial instability, autism spectrum disorder, epilepsy, cerebrovascular disease including moyamoya syndrome and amyloid angiopathy, and Alzheimer’s disease [5]. This case report presents three cases of suspected PTU in patients with Down syndrome at one primary care center in the last 4 years.

## 2. Case Presentations

### 2.1. Patient 1

Patient 1 is a 2‐year‐old African American female born preterm at 35 weeks and 3 days via spontaneous vaginal delivery to a G6P3 34‐year‐old mother. Pregnancy was complicated by suspected Trisomy 21, with fetal echocardiogram concerning for a left superior vena cava (SVC), and maternal Type 2 diabetes. Delivery was complicated by respiratory distress initially requiring continuous positive airway pressure (CPAP), and the patient was discharged home after staying in the Neonatal Intensive Care Unit (NICU) for 4 days. She presented to the Emergency Department (ED) at 2 months of age with concern for unresponsive episodes characterized by staring, pallor, and spitting up with backarching. These symptoms would last for 3‐4 min. These episodes were later attributed to gastroesophageal reflux. She presented again at 13 months of age for episodes during which her whole body stiffened, and her eyes rolled upwards for 1‐2 s. She had no associated loss of consciousness or postictal phase. The patient’s mother reported an average of 3 episodes per week, often associated with being excited or upset.

The patient was admitted at the time of both presentations and underwent two video EEGs (vEEGs) which were normal for her age, showing symmetric, continuous, and synchronous background cerebral activity in both hemispheres. There were no noted electrographic or electroclinical seizures, and no epileptiform discharges identified. It is, however, not documented whether the episodes of concern were witnessed during the two studies. The first EEG lasted approximately 14 h and included both the awake and sleep states. The second EEG lasted for 75 min and was also obtained during wakefulness and sleep. No head imaging was obtained during her evaluations. The patient was initiated on famotidine for presumed gastroesophageal reflux disease (GERD) given her reported spit ups with associated backarching during episodes. However, the second episode was ultimately attributed to PTU (Table 1).

**TABLE 1:** The similarities and differences between three pediatric patients who share a history of Down syndrome who presented with similar episodes and each underwent reassuring neurologic evaluations.

	Patient 1	Patient 2	Patient 3
Sociodemographic	2 years old	2 years old	4 years old
	African American	Caucasian	Caucasian
	Female	Male	Female

Relevant history	Down syndrome	Down syndrome	Down syndrome
	Preterm (35 weeks)	Di‐di twin	OSA
	Left SVC	GERD	Constipation
		Laryngomalacia	Congenital cardiac defects
		RAD	
		Bilateral hypermetropia	

Presenting symptoms	Body stiffening	Stiffening of extremities	Stiffening of extremities
	Staring episodes with pallor	Eyes rolled backwards	Eyes rolled backwards
	Eyes rolled backwards	Facial grimacing	Unresponsive episode
		Association with feeds	Laceration secondary to above activity

Evaluation	vEEG (x2)	CT head w/o contrast vEEG	vEEG

EEG findings	#1: No epileptiform discharge, electrographic, or electroclinical seizure.	Mild generalized background slowing.	Some focal spike‐wave discharges in sleep, but none in awake state.
	#2: No significant asymmetries, slowing, or epileptiform discharges.	No epileptiform discharges, electrographic, or electroclinical seizures.	
		One push button event, no electrographic correlate.	

Neuroimaging findings	N/A	No acute intracranial abnormality, bilateral chronic sinusitis, or bilateral otitis media.	N/A

Duration of follow‐up	Ongoing (> 3 years)	Ongoing (> 2 years)	Ongoing (> 1 year)

Clinical outcome	Self‐resolution of symptoms		Ongoing episodes

*Note:* All three patients were ultimately diagnosed with PTU. Symptoms have since self‐resolved for two of the three patients.

### 2.2. Patient 2

Patient 2 is a 2‐year‐old Caucasian male who was born at 38 weeks and 2 days via Cesarean section due to breech presentation to a 27‐year‐old G1P1 mother, with complication of di–di twin pregnancy. Delivery was uncomplicated. The patient required a 3‐day NICU stay for respiratory distress requiring CPAP prior to discharge home. He was diagnosed with Down syndrome while hospitalized. During his early months, he was diagnosed with GERD, laryngomalacia, bilateral hypermetropia, torticollis, plagiocephaly, and global developmental delay. At 15 months of age, the patient presented to the ED with the complaint of seizure‐like activity during an outpatient speech therapy session. While strapped into a highchair, he suddenly became unresponsive, his eyes rolled back, and he had associated facial grimacing and stiffening with extension of his extremities (Video 1). Episodes continued intermittently for approximately 5 min, after which the patient returned to baseline. He presented again shortly thereafter for daily clusters of rapid, forced eye closure, forward head jerking, and eyes rolling backwards, lasting 1–3 s per episode. Clusters of episodes would last 5–10 min.

Labs including complete blood count (CBC), comprehensive metabolic panel (CMP), and urine toxicology were obtained and unremarkable. CT head without contrast was obtained and normal. vEEG monitoring was subsequently performed for 19 h, during both periods of wakefulness and sleep, showing no epileptiform abnormalities correlated to stereotyped behavior that occurred during study. The stereotyped behavior that prompted the singular push‐button event consisted of bilateral arm twisting and shuddering with the patient’s head down. There was, however, mild generalized background slowing noted on vEEG, consistent with diffuse cerebral dysfunction, that was noted to be consistent with the patient’s history of Down syndrome. There were no interhemispheric voltage, frequency asymmetries, or epileptiform discharges recorded. Episodes were attributed to PTU (Table 1). Episodes continued to be associated with feeds. He continued to work with speech therapy and remained on antireflux medication. Episodes have since self‐resolved.

### 2.3. Patient 3

Patient 3 is a 4‐year‐old Caucasian female who was born at 38 weeks via repeat Cesarean section to a 34‐year‐old G2P1 mother with pregnancy complicated by ultrasound revealing fetal anomalies including complete balanced atrioventricular (AV) canal and pyelectasis, with genetic noninvasive prenatal testing (NIPT) confirming Trisomy 21. Delivery was complicated by nuchal cord and meconium‐stained fluid, requiring CPAP. The patient required a 35‐day NICU stay to rule out sepsis, as she had leukopenia on initial labs, as well as thrombocytopenia, difficulty feeding, hypoglycemia, and respiratory distress. Echocardiograms during NICU admission revealed congenital cardiac anomalies including AV canal defect and vascular ring, now status post repair. At 3 years of age, the patient presented to the ED for concern of episodes characterized by stiffening of both arms and legs, eyes rolling back, and falling forward—resulting in a chin laceration. As documented, the episode reportedly lasted 15–20 s, with a subsequent 5–10 min period of confusion. A similar episode had occurred once before, about a week prior to presentation. Of note, the patient’s mother has a history of epilepsy. The patient appeared to be unresponsive during one of the episodes; however, this was not a consistent finding across her episodes.

A vEEG was performed that ran overnight, showing left central vertex epileptiform discharges during sleep, although no corresponding events were noted, and changes were not present in the awake state. The neurologist interpreted these changes as abnormal but not suggestive of epilepsy. No head imaging was obtained. Neurology recommended monitoring without antiseizure medications, with repeat vEEG if events reoccur. Episodes self‐resolved at that time, and no further vEEG studies were performed. The reported confusion following the patient’s episode was attributed to fatigue rather than a postictal state. Episodes were clinically considered consistent with PTU (Table 1).

When recalling these episodes later, the patient’s mother did not recall her daughter stiffening during the episode, loss of consciousness, or the presence of a postictal state. She states that the patient was alert and active subsequent to her fall. However, she did recall the fall resulting in a chin laceration. The patient would appear to blink in order to stop the episodes when they occurred. The patient’s mother also describes that the patient does experience conjugate upward deviation of the eyes, characteristic of PTU. Although initially thought to have completely self‐resolved, the patient’s mother reports that these episodes continue to occur.

## 3. Discussion

The three pediatric patients described above with Down syndrome presented with similar episodes involving stiffening of their extremities, upward eye movements, and altered responsiveness lasting several seconds before returning to baseline. Of note, all three patients preserved consciousness during episodes, with the exception of brief questionable unresponsiveness during one of Patient 3’s episodes—however, this was denied by the patient’s mother during a subsequent encounter. A vEEG was obtained in each case, as EEG findings are critical for distinguishing between PTU and seizures. The EEG findings in the above cases did not suggest that the episodes that the patients were experiencing were seizures. However, it should be noted that for Patient 1, it was not documented whether an episode occurred during EEG study; and for Patient 3, it was noted that no events took place during study. For Patient 3, abnormal activity was present on vEEG although it was not correlated to clinical findings or thought to be suggestive of epilepsy. Seizures were carefully considered on the differential diagnosis of what was causing Patient 3’s episodes, especially given the brief questionable episode of unconsciousness and the noted chin laceration. In addition, infantile spasms are an important consideration and may also be missed if the episode is not captured during a patient’s EEG. Discussion was had as to whether antiseizure medication should be initiated, the EEG repeated at a later time, or if the patient should be monitored off of medication. Ultimately, due to the clinical appearance of the episodes as reported by the patients’ family and primary care provider, the appearance of the vEEG as interpreted by the Neurology team, and the apparent self‐resolving nature of symptoms, seizures were determined to be less likely and the patient was monitored off of antiseizure medication. If symptoms were to have returned later in this patient’s clinical course, or if she were to have a change in the appearance of her episodes, the diagnosis of seizures would need to be revisited. While episodes have more recently occurred as subsequently reported by the patient’s mother, they have not appeared consistent with seizure‐like activity or involved the loss of consciousness.

In addition, head imaging was not obtained for each of the above patients during their neurologic evaluation as it was not deemed to be clinically indicated. For Patient 1, because she had two reassuring EEGs and clinical findings that were not suggestive of a focal neurological defect, it was decided that neuroimaging would not change management. Similarly, for Patient 3, her neurologic exam was nonfocal, and because symptoms resolved without medication, imaging was not deemed necessary at that time. Given the appearance of the patients’ episodes, and the lack of more likely diagnoses, the patients above were diagnosed with PTU rather than epilepsy. However, if episodes were to return, further workup including prolonged vEEG may be warranted.

The pathophysiology of PTU is currently unknown, but some cases with similar findings have identified the upper dorsal brainstem as a likely site of physical disturbance [2]. However, the intermittent nature and lack of identification of structural lesions have led some to postulate that the episodes might be due to either the immaturity or depletion of a neurotransmitter or an unrecognized channelopathy [2]. Other theories include immature corticomesencephalic control of vertical gaze and failure of cortical compensation during stressors or a cerebellar disorder [3]. These ideas connect with our suspected increased prevalence in patients with Down syndrome because these patients often have altered synaptic transmission, decreased neurogenesis, reduced plasticity, and other aberrant processes [6].

A differential diagnosis for patients presenting with PTU‐like symptoms should always include epilepsy, which is more common in patients with Down syndrome than the general population [6]. Epilepsy is often overdiagnosed in children, so a thorough evaluation should be completed to determine the diagnosis. PTU is different from epilepsy in that it is limited to eye symptoms, has no postictal period, and has a lack of spike activity on EEG during the upgaze episodes [2]. It should be noted that individuals with Down syndrome without epilepsy have been shown to have elevated slow wave activity and abnormalities in the alpha and beta frequency bands on EEG [7, 8]. Other causes of upward gaze deviation include lesions in the medial longitudinal fasciculus resulting in sustained tonic upward gaze, parkinsonian features, drug crisis, coma due to cardiac causes, brain tumors, and ocular tics that include voluntary upgaze [2]. PTU is a diagnosis of exclusion, requiring both the characteristic symptoms and EEG results that are not suggestive of epilepsy.

In general, PTU is thought to be a benign condition that will usually resolve on its own within several years. Kartal [1] reported that most episodes stop between one and four years of onset without any therapy or treatment. Of note, Patient 3 is still within the one to four year window during which symptoms are expected to resolve. Treatment with anticonvulsants, acetazolamide, and adrenocorticotropic hormone (ACTH) have been shown to have no benefit and are not recommended [2]. Individual patients have been noted to improve on levodopa, but routine treatment with levodopa is not recommended [1]. Therefore, it is important to differentiate PTU from epilepsy in patients with a history of Down syndrome to prevent the use of antiepileptic medications when not, otherwise, indicated and, therefore, eliminate the risk of associated side effects. PTU is thought to be associated with developmental delay in more than half of the patients although we did not identify any new developmental delay in these patients at the time of this study. However, we will continue to monitor for increased prevalence of severe developmental delay in these patients. Autism and intellectual ability are more prevalent in patients with Down syndrome, but it is possible that they could be seen at further increased rates in those also diagnosed with PTU.

The cases described above are similar in that all three patients have a history of Down syndrome and early in life presented with seizure‐like activity with, otherwise, normal neurologic workup including unremarkable vEEG and several also with normal head imaging. These results are less suggestive of epilepsy and suggest instead stereotypic behaviors consistent with PTU. In all the above cases, management included anticipatory guidance, including the recommendation to pursue further evaluation in the case of worsening of episodes or change in character, as well as seizure precautions. Patients also are receiving therapies for other medical conditions as appropriate, including Speech, Occupational, and/or Physical Therapy.

Limitations of this retrospective case report include variability between the evaluations that each individual patient underwent while underlying epilepsy was being considered, including length and quantity of vEEGs, occurrence of events during vEEG evaluation, availability of head imaging results, and subspecialist evaluation. Two of the described patients did not undergo head imaging, so we cannot definitively say that a structural abnormality was not causing these episodes. Head imaging was not obtained due to a variety of reasons, including resolution of symptoms, low suspicion for abnormal anatomy, and shared decision making. A lack of head imaging fails to rule out an anatomical cause for a PTU‐like presentation, but unfortunately, this imaging cannot be obtained retrospectively. Our retrospective assessment of the patients described is also limited by the documentation available that describes each patients’ clinical presentation. Ocular motor findings consistent with PTU such as neck flexion and downbeating saccades were reportedly observed by the patients’ families and their primary care provider. However, these specific findings are not clearly documented within the patients’ charts, and we are, therefore, unable to strengthen our study by including these findings. In addition, ophthalmology was not consulted to evaluate the patients described here. This study is also limited in that these patients’ clinical courses are ongoing, and alternative etiologies or confounding variables may become apparent in the future.

Nevertheless, it is important to consider PTU as a potential etiology in pediatric patients with a history of Down syndrome presenting with seizure‐like activity, and we hypothesize that it could be more prevalent in this patient population. These cases suggest that PTU should be considered as a possible diagnosis in seizure‐like episodes in children with Down syndrome. This case report serves to raise awareness of a possible association between PTU and Down syndrome and to recognize the need for future studies to better understand the prevalence of this association.

## Funding

This work was supported by the research department at Loma Linda University School of Medicine.

## Consent

Written consent to publish has been obtained by the parents of the patients described.

## Conflicts of Interest

The authors declare no conflicts of interest.

## Supporting Information

Additional supporting information can be found online in the Supporting Information section.

## Supporting information


**Supporting Information** Video 1. Included in our submission is a video clip demonstrating Patient 2 experiencing an episode of abnormal movement that prompted presentation to ED. The episode is characterized by his eyes rolling back, facial grimacing, and stiffening with extension of his extremities.

## Data Availability

The data that support the findings of this study are available from the corresponding author upon reasonable request.
